# Measuring the effect of the anti-nerve growth factor antibodies bedinvetmab and frunevetmab on quality of life in dogs and cats with osteoarthritis using a validated health-related quality of life outcome measure: an observational real-world study

**DOI:** 10.3389/fvets.2024.1395360

**Published:** 2024-08-14

**Authors:** Jacqueline Reid, Edwina Gildea, Vinny Davies, Jill Thompson, Marian Scott

**Affiliations:** ^1^School of Biodiversity, One Health and Veterinary Medicine, University of Glasgow, Glasgow, United Kingdom; ^2^Zoetis (Ireland), Dublin, Ireland; ^3^School of Mathematics and Statistics, Glasgow, Glasgow, United Kingdom

**Keywords:** dogs, cats, health-related quality of life (HRQL), VetMetrica, bedinvetmab, frunevetmab, real world study, smartphone app

## Abstract

**Background:**

Osteoarthritis causes chronic pain, impaired joint function, decreased mobility and negatively impacts quality of life (QOL). Anti-nerve growth factor antibodies bedinvetmab for dogs and frunevetmab for cats are licensed for the alleviation of osteoarthritis pain but their QOL impact is unreported. Our aim was to determine if these therapeutics improve QOL using a validated health-related QOL measure that generates scores in four domains of QOL-energetic and enthusiastic (E/E), happy and content (H/C), active and comfortable (A/C) and calm and relaxed (C/R)-in the dog and three in the cat-vitality, comfort and emotional wellbeing (EWB). Summary scores for physical wellbeing (PWB) and emotional wellbeing (EWB) for dogs and PWB for cats are calculated from the domain scores.

**Methods:**

Animals received bedinvetmab (dogs) at 0.5–1 mg/kg or frunevetmab (cats) at 1–2.8 mg/kg by subcutaneous injection on days 0, 28 and 56 and owners completed QOL assessments within 48 hours of day 0 and on days 14, 28, 56, 63 and 70 using a study-specific app.

**Results:**

Assessments were completed by 75 dog and 56 cat owners. By day 14 there was a statistically significant improvement (*p* ≤ 0.001) in PWB, EWB and all domains except C/R (*p* = 0.005) in dogs and in all domains and PWB in the cat. Subsequently there was a continued improvement in all domains and summary scores (*p* ≤ 0.001) except for H/C in the dog and EWB in the cat, which were excluded from the statistical model. The overall improvement in all domain scores in the cat and E/E and A/C in the dog exceeded the previously reported minimum important difference scores for the QOL measure, indicating a clinically significant change.

**Conclusion:**

Treatment with bedinvetmab and frunevetmab produced a significant improvement in the QOL of dogs and cats. This latest evidence for the use of these OA pain medications could be helpful in the clinical management of osteoarthritis and post-marketing clinical trials.

## 1 Introduction

Osteoarthritis (OA), a degenerative condition that causes chronic pain, loss of joint function and decreased mobility, affects an estimated 20–37% of dogs aged >1 year (1–3), with higher prevalence recorded in prospective investigations compared with retrospective reviews where OA is confirmed only on the basis of its mention in the clinical record (4). Furthermore, studies have shown that 64–90% of cats have radiographic changes relating to degenerative joint disease (DJD) with approximately 45% of these cats having pain with clinical signs related to impaired mobility (5, 6).

Osteoarthritis causes chronic pain, impaired joint function, decreased mobility and negatively impacts quality of life (QOL). This is not surprising as it has also been shown that chronic pain has a broad negative impact on many aspects of health including affect, sleep, function, cognition and social relationships (7). People with OA have reported that the condition causes significantly lower levels of quality of life (QOL) and in their paper “Global management of patients with knee osteoarthritis begins with quality of life assessment: a systematic review,” Vitaloni et al. (8) emphasise that QOL data is a valuable tool, providing clinicians with a better understanding of OA to facilitate implementation of the most effective management plan.

Veterinarians in the UK rate OA to be the most common cause of chronic pain in dogs (9), and so it is not surprising that there is a growing body of evidence that the disease negatively impacts the QOL of affected animals (10–12). Furthermore, since the goal of treatment of OA is to improve the overall QOL by relieving joint pain, delaying progression of the disease, and restoring mobility, it is important to be able to measure the effect on QOL of any therapeutic used as part of multimodal therapy (13). When asked in a survey “How important are the following when choosing a product for the alleviation of pain due to osteoarthritis in dogs,” 900 European veterinarians rated “improves the QOL of dogs with OA” highest in a list which included “effectively reduces OA pain” and “improves mobility.”^1^

Non-surgical options for OA treatment in companion animals include a multimodal approach involving lifestyle changes, weight management, physiotherapy and medical management of pain, the mainstay of which is therapy with non-steroidal anti-inflammatory drugs (NSAIDs). However, not all animals respond to NSAIDs and in some cases they are not well tolerated in dogs (14, 15) and cats (16, 17). Furthermore, when used alone NSAIDs are insufficient to control the pain of OA in many cases (14, 18). To address the requirement for more effective and better tolerated therapeutics for OA pain, research has been conducted on alternative therapeutic targets such as nerve-growth factor (NGF). For several decades preclinical and clinical studies have shown that NGF plays a notable role in rodent models and human chronic pain states, including that of OA (19, 20). A review article by Enomoto et al. (21) stated that “anti-NGF therapy looks to be both very effective and very promising as a novel therapy against chronic pain in dogs and cats”. Subsequently, the European Commission authorized the use of the anti-NGFmAb bedinvetmab (approved as Librela in the European Union in 2020) for the alleviation of osteoarthritis pain in dogs^2^ and the anti-NGF mAb frunevetmab (approved as Solensia in 2021 in the European Union & the UK) for cats with OA.^3^ Since approval in Europe, these two products have been approved for use in many countries across the world. The efficacy of these therapeutics has been reported in dogs by Corral et al. (22) and in cats by Gruen et al. (23) using the following clinical metrology instruments-the Canine Brief Pain Inventory (CBPI)^4^ in dogs and the Client Specific Outcome Measures (CSOM) (24) in cats-as primary outcome measures. These instruments are owner-reported questionnaires primarily designed to measure the functional impairment caused by the disease. Although the CBPI contains one global question for the owner which asks them to quantify their dog’s QOL, such global questions are subject to response bias and cannot be considered validated QOL measures (25). The aforementioned studies, carried out for regulatory purposes and to provide veterinarians with data on product efficacy and safety at launch, were conducted in controlled conditions in a population limited by strict inclusion and exclusion criteria and a well-defined protocol. Conversely, in studies conducted under real-world conditions (RWD) the clinician’s decision to prescribe the medication, consistent with approved prescribing information and in accordance with clinical practice, precedes the decision to enrol the animal in the study. Furthermore, there are no additional diagnostic tests or visits beyond that which is consistent routine clinical practice. The way in which a drug performs in real-world conditions has been described as a test of its effectiveness (26).

According to the FDA “Real-world data are data relating to patient health status and/or the delivery of health care routinely collected from a variety of sources, the purpose of which is to produce real-world evidence (RWE), defined as the clinical evidence about the usage and potential benefits or risks of a medical product.” Examples of RWD include data derived from electronic health records, medical claims data, data from product or disease registries, and data gathered from other sources (such as digital health technologies) that can inform on health status” (27). Digital health technologies include patient-generated health data from internet-based tools, passively (for example via an activity monitor) or actively by the patient entering data on a web-based questionnaire. Importantly, sources of RWD are observational, meaning that any interventions are not determined by a study protocol, but are decided and instigated by the attending clinician (27).

Quality of life is a broad concept which covers all aspects of life whereas health-related quality of life (HRQL) has a focus on the effects of illness and the impact its treatment may have on QOL. Health-related quality of life instruments can be specific, focusing on individual conditions (disease-specific), or they can be generic, designed for use in a variety of contexts. VetMetrica^™^ is an umbrella term which covers two behaviour-based HRQL instruments, one for the dog (28–31) and the other for the cat (32). These are generic HRQL structured questionnaire instruments with a formal scoring methodology, completed online by the owner in around 5 min. Although disease-specific instruments may be more sensitive to clinical change, generic instruments are useful to quantify a range of impacts related to disease and its treatment and may be the only choice when a patient has more than one condition, a situation encountered commonly in veterinary medicine (32, 33). Generic instruments can generate a single item score or a profile of scores which reflect an individual’s health status on multiple domains of HRQL, with each domain being allocated a score. This allows visibility of the impact of interventions on different elements of HRQL and may increase the sensitivity to changes in health status (34). Generic instruments have been shown to perform well in several disease states in people including OA (35) and VetMetrica^™^ instruments for the dog and cat have been shown to be responsive to clinical change in dogs (36, 37) and cats (38) with OA.

To date, no studies to determine the impact of bedinvetmab or frunevetmab on QOL of dogs and cats respectively have been reported using a validated HRQL measure. The purpose of this investigation was to generate RWD to investigate whether bedinvetmab and frunevetmab improve the QOL of dogs and cats with OA, using the previously validated HQRL outcome measure VetMetrica^™^.

## 2 Materials and methods

### 2.1 Study design

This investigation used a subset of HRQL data (Supplementary material S1) collected during two species-specific, multi-centre, non-interventional, uncontrolled, prospective, longitudinal, field studies designed to evaluate animal and pet owner QOL and pet owner treatment satisfaction in dogs and cats in the UK, treated with bedinvetmab and frunevetmab respectively. These studies were reviewed and approved by the Zoetis Ethical Review Board. Data were collected using a study-specific app developed by Celeritas Digital (New York, United States) and electronic informed consent was given via agreement to Terms of Use & Privacy information in the app, a copy of which was automatically emailed to the owner on agreement.

The app recorded owner name, email address and telephone number, pet name, breed in the case of dogs, sex, date of birth and bodyweight, veterinary clinic and bedinvetmab/frunevetmab injection dates. VetMetrica^™^ questions for the owner were programmed into the app and owner responses to these were encrypted and submitted securely, via a SOAP based web service, to the VetMetrica^™^ server along with a unique identifier for the animal. Computation and reporting of scores back to the app was instantaneous and automatic.

### 2.2 Measurement of HRQL

VetMetrica^™^ structured questionnaires comprise 22 behaviour-based items for the dog owner (31) and 20 for the cat owner (32), completed online in around 5 min (Supplementary material S2). These items are simple descriptive terms, which are either positive (words associated with health) or negative (words associated with ill health). Each item is associated with a 7-point (0–6) scale which allows the owner to rate the extent to which the term describes their dog or cat’s behaviour. For example, for the term “playful,” 0 represents “not at all playful” and 6 represents “could not be more playful.” So, in the case of a positive item like “fun-loving” a score of 6 implies very good HRQL, but the same score implies very poor HRQL when the item is negative, for example “depressed.” It is important to note that unlike other instruments where a total HRQL score is obtained by simple addition of the owner ratings for each item, in the case of VetMetrica^™^, a coded algorithm automatically transforms the owner responses into scores (0–6) in 4 domains of QOL-energetic/enthusiastic (E/E), happy/content (H/C), active/comfortable (A/C), calm/relaxed (C/R)-for the dog and 3 domains-vitality, comfort and emotional wellbeing (EWB)-for the cat. To assist with interpretation, these domain scores are then normalized to the healthy dog or cat population, so that a score of 50 represents the score for the average healthy dog (39) or cat (40). Additionally, the normalization is such that 70% of healthy dogs or cats will score above a threshold value at 44.8. In the dog, the domains E/E and A/C are made up of items that represent physical wellbeing (PWB) and the items making up H/C and C/R represent emotional wellbeing (EWB). Summary scores for PWB and EWB are calculated by averaging scores in the relevant domains. Similarly, in the cat Vitality and Comfort represent PWB for which a summary score can be calculated in the same way.

Responsiveness to clinical change for both species was quantified previously by calculating the minimum important difference (MID), which is defined as “the smallest difference in score in the outcome of interest that informed patients or informed proxies perceive as important, either beneficial or harmful, and which would lead the patient or clinician to consider a change in the management” (41). The MID for the dog with non-specific chronic disease is 7 for all 4 domains (39) and similarly for the cat it is 5, 7.5 and 5 for the domains vitality, comfort and emotional wellbeing respectively (40).

### 2.3 Study sites and personnel

A team of 6 Zoetis veterinary consultants recruited 27 first opinion general veterinary practices, located across Scotland, Northern Ireland, Wales & England to participate in the canine study and 24 practices across the same locations in the feline study. Details of the study background and protocol were explained to the practice clinicians. There was no obligation for the practice to participate and clinicians were not paid to recruit cases. Following diagnosis of OA and only if a decision had been made to prescribe bedinvetmab for the canine patient or frunevetmab for the feline patient, the clinician provided the pet owner with an information leaflet detailing the study. It was the decision of the pet owner whether they wished to participate in the study and move forward with downloading the study app. If they chose to be involved, they were reimbursed for the time taken to complete the 6 assessments over the 70-day trial period, but not for their animal’s treatment.

### 2.4 Subjects

Client owned dogs and cats ≥12 months of age, diagnosed with OA according to the attending clinician’s standard clinical practice, being prescribed bedinvetmab (dogs) or frunevetmab (cats) for the alleviation of pain in accordance with the European Summary of Product Characteristics (SPC) and who had not been prescribed alternative pain medications, such as NSAIDs, opioid analgesics and systemic corticosteroids, in the previous 2 weeks were eligible for the study.

### 2.5 Study timeline

On day 0, eligible animals received bedinvetmab (dogs) at the licensed dose of 0.5–1 mg/kg or frunevetmab (cats) at 1–2.8 mg/kg by subcutaneous injection. Their owners were given an information sheet explaining the appropriate field study, according to whether their animal was a dog or cat and were invited to download the study-specific app if they agreed to participate in the study. The app invited them to complete the first VetMetrica^™^ assessment for their animal within 48 h of day 0 to ensure their participation in the trial. The data from animals whose owners did not comply with this deadline were excluded from the analysis. Subsequent injections of bedinvetmab and frunevetmab were administered at 28-day intervals on days 28 and 56. Injection reminders took the form of push notifications via the app at 21 days following the preceding injection.

**Figure 1:**
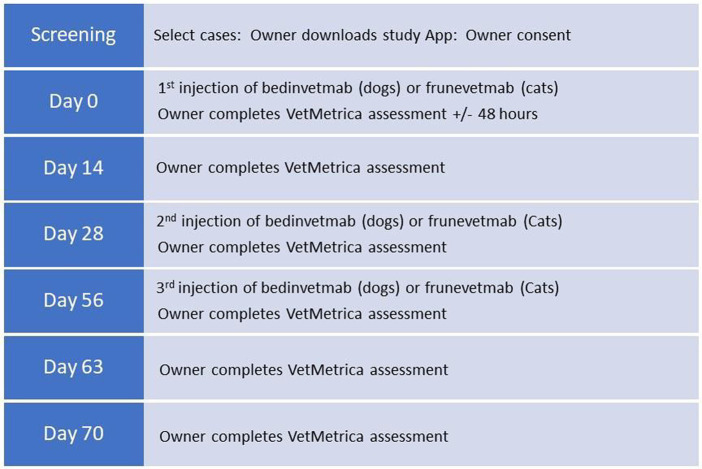
Study timeline.

Owners were requested to complete additional VetMetrica^™^ assessments on days 14, 28, 56, 63 and & 70. Assessment push notifications via the app were set as follows: questionnaire due date, +1 day, +5 days. After 6 days from the due date assessments expired. Graphical representation of the timeline is shown in Figure 1.

### 2.6 Statistical methods

Data were analysed using Minitab^®^ version 20 Statistical Software (2010)^5^ and with an open-source statistical software environment (R, version 4.3.1, R Foundation for Statistical Computing, Vienna, Austria). The level of statistical significance was set at 5% (*p* < 0.05) for all analyses.

Exploratory analysis of the scores was carried out using box plots. Thereafter, for formal analysis, HRQL scores were first converted into change in HRQL scores. For each animal and domain, the first score was subtracted from the others, such that the 6 original scores were converted into 5 measures of change in the HRQL domain. Regression models were then fitted for each animal domain where terms could include fixed effects for the intercept (the initial change in score from day 0 to day 14) and time (the change from day 0 to days 28, 56, 63, 70), and a random effect for the slope which takes account of the fact that animals will change in a domain at different rates to each other. No random effect intercept term was considered as this was accounted for by converting the original scores to change scores. Four possible models were fitted and considered for the analysis: (1) intercept only (2) intercept and time component (3) intercept and random slope and (4) intercept, time component, and random slope. Models were compared using the Bayesian information criteria (BIC), with the model with the lowest BIC selected as the best model for each combination of species and domain.

## 3 Results

### 3.1 Subjects

**Figure 2:**
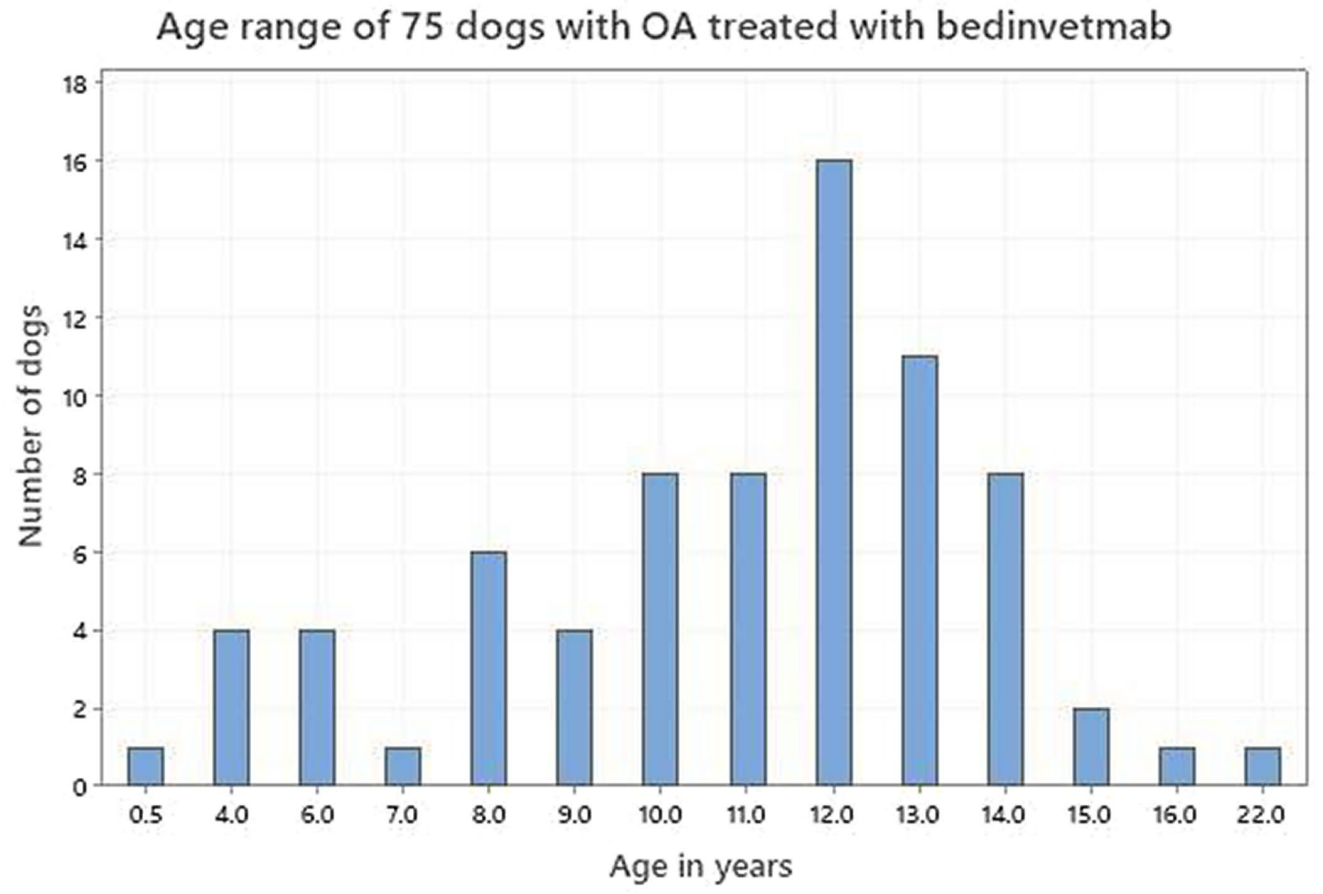
Age range of 75 dogs with osteoarthritis treated with bedinvetmab.

**Figure 3:**
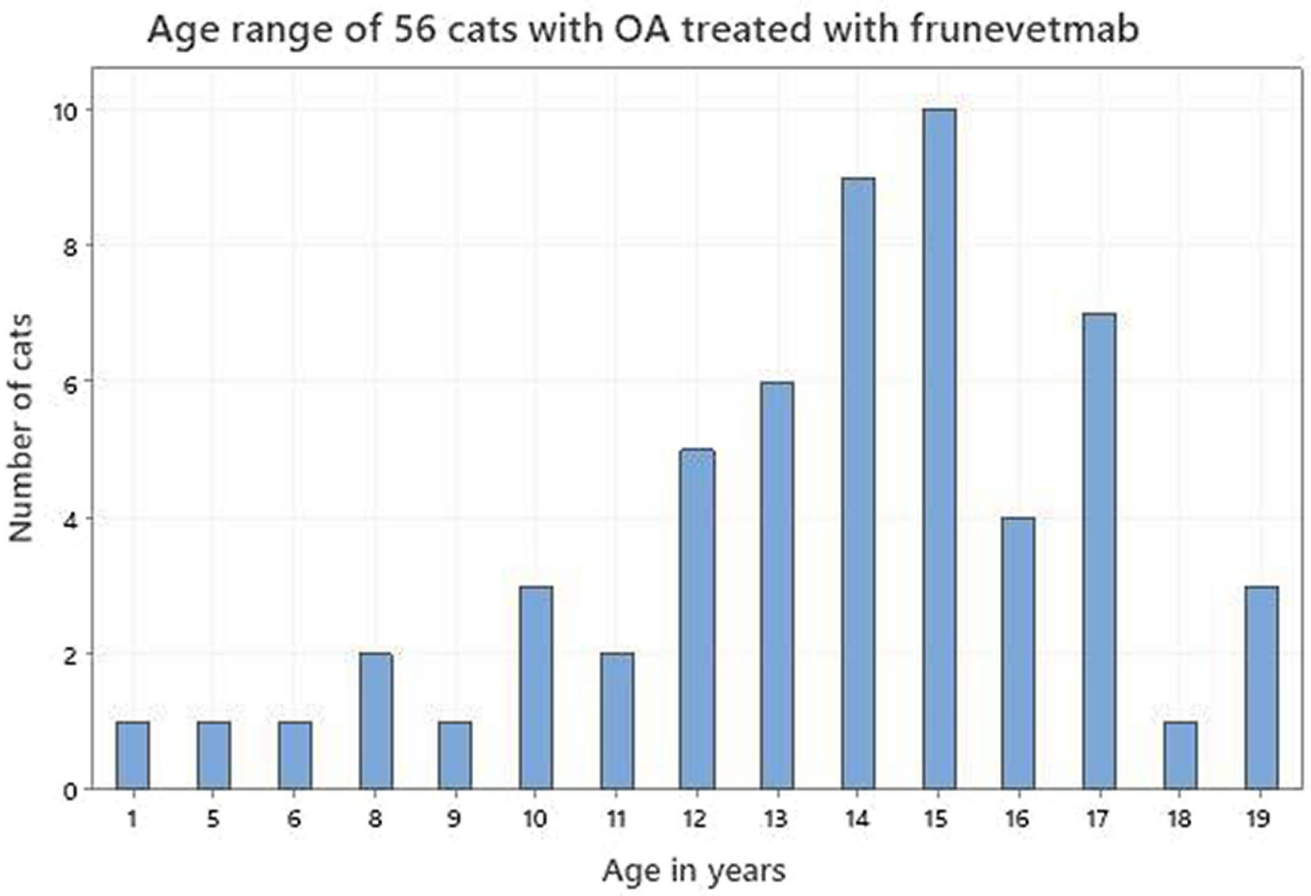
Age range of 56 cats with osteoarthritis treated with frunevetmab.

Seventy-five dog owners and 56 cat owners completed assessments in accordance with the study protocol. The demographics of both dogs and cats are shown in Table 1. Figures 2, 3 depict the age range of dogs and cats, respectively. There were 22 dog breeds represented (Table 2) with Labrador retrievers and crossbreeds predominating. There were no giant breed dogs in the sample, but 22 dogs were classified by breed as large (29%). Furthermore, 56, 27 and 17% of the sample population were classified by weight as large (21–50 kg), medium (11–20 kg) and small (≤10 kg). Cat breeds were not recorded.

**Table 1:** Demographics of dogs and cats treated with bedinvetmab (dogs) and frunevetmab (cats).

	Dogs (*n* = 75)	Cats (*n* = 56)
**Age in years**		
Mean ± SD	10.9 ± 3.3	13.6 ± 3.5
Median (range)	12 (0.5–22)	14 (1–19)
**Weight (kg)**		
Mean ± SD	22.9 ± 11.3	4.5 ± 0.9
**Size**		
Small ≤10 kg	13	N/A
Medium 11–20 kg	20	
Large 21–50 kg	42	
**Sex**		
Entire male	15	6
Neutered male	18	24
Entire female	7	6
Neutered female	35	20

SD, standard deviation.

**Table 2:** Number of times breeds were represented in dogs treated with bedinvetmab.

Breed	Number
Labrador Retriever	16
Crossbreed	16
Staffordshire Bull Terrier	6
Border Collie	5
Cocker Spaniel	4
Unspecified	4
Golden Retriever^a^	3
Chihuahua	3
Flat Coat Retriever^a^	2
Jack Russell Terrier	2
Shi Tzu	2
Border Terrier, Cockapoo, Greyhound	1
English Pointer, Irish Terrier, Labradoodle	
Maltese Terrier, Poodle, Pug, Rottweiler^a^	
Schnauzer, Siberian Husky	

a Represents those breeds classed as large by the UK Kennel Club.

#### 3.1.1 Dogs

**Figure 4:**
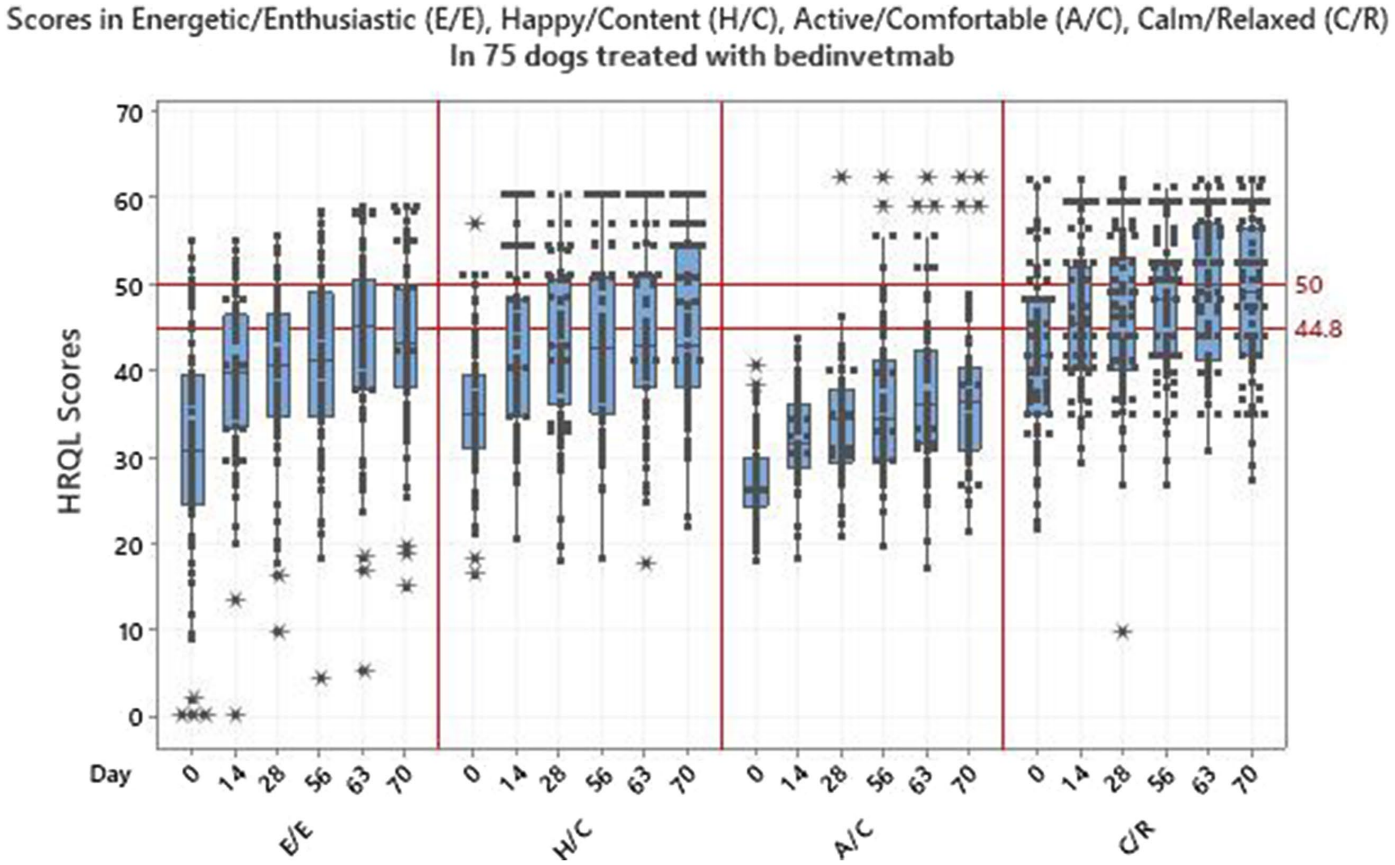
Scores in 4 domains of QOL-energetic/enthusiastic, happy/content, active/comfortable, calm/relaxed-in 75 dogs with osteoarthritis treated with bedinvetmab.

Table 3 shows the HRQL descriptive statistics for the 75 dogs treated with bedinvetmab and Figure 4 shows the HRQL scores over time in all four domains. There was marked variability in the scores in all domains although this was less evident in the A/C domain. Furthermore, at day 0 the median score for this domain was considerably lower than those of the other domains which were, nevertheless, below the threshold above which 70% of healthy dogs will score. For E/E, H/C and A/C, the median scores did not reach the 44.8 threshold, whereas for C/R all scores from day 14 were between 44.8 and 50, the latter representing the average healthy dog.

**Table 3:** Descriptive statistics for scores in each of 4 domains of QOL (energetic/enthusiastic, happy/content, active/comfortable, calm/relaxed) and 2 summary scores physical wellbeing (PBW) and emotional wellbeing (EWB) at baseline (day 0, within 48 h of initial treatment with bedinvetmab) and 5 subsequent time points to day 70 in 75 dogs with osteoarthritis.

Domain	Day	Mean ± SD	Median	Q1	Q3	IQR
Energetic/enthusiastic	0	30.73 ± 12.44	30.53	24.45	39.28	14.83
	14	38.75 ± 9.56	39.83	33.24	46.41	13.17
	28	39.53 ± 9.45	40.65	34.47	46.45	11.98
	56	41.00 ± 10.57	41.25	34.50	40.02	14.52
	63	43.02 ± 10.86	45.01	37.85	50.49	12.64
	70	43.08 ± 9.52	43.17	37.93	49.41	11.48
Happy/content	0	35.37 ± 8.09	34.86	30.89	39.41	8.52
	14	41.65 ± 9.33	40.22	34.50	48.19	13.69
	28	42.14 ± 9.27	42.71	35.94	50.46	14.52
	56	43.15 ± 9.97	42.62	36.94	50.83	15.89
	63	44.56 ± 10.38	42.84	38.09	51.02	12.93
	70	44.57 ± 9.74	42.88	38.03	54.47	16.44
Active/comfortable	0	27.30 ± 4.91	26.46	24.17	29.77	5.6
	14	32.48 ± 5.26	31.49	28.75	35.91	7.16
	28	34.00 ± 6.19	34.25	28.28	37.71	8.43
	56	36.67 ± 8.73	34.38	29.49	41.13	11.64
	63	37.53 ± 9.20	36.15	32.50	42.24	10.74
	70	37.08 ± 8.48	36.31	30.77	40.24	9.47
Calm/relaxed	0	41.66 ± 9.27	41.71	34.91	48.25	13.34
	14	45.87 ± 7.82	45.38	40.27	51.83	11.56
	28	45.82 ± 9.34	46.30	40.05	53.08	13.03
	56	47.44 ± 8.00	48.25	41.71	52.53	10.82
	63	49.49 ± 8.36	49.80	41.01	56.98	15.97
	70	48.34 ± 8.96	48.95	41.71	56.25	15.54
Physical wellbeing (PWB)	0	29.02 ± 7.61	28.50	24.95	34.43	9.48
	14	35.62 ± 6.64	35.50	31.73	40.92	9.19
	28	36.76 ± 7.18	37.46	32.10	41.41	9.31
	56	38.83 ± 8.79	37.94	32.51	44.44	11.93
	63	40.28 ± 9.36	41.09	34.81	46.62	11.81
	70	40.08 ± 8.29	39.67	34.45	45.13	10.68
Emotional wellbeing	0	38.52 ± 7.41	37.50	33.64	43.54	9.9
	14	43.76 ± 7.24	42.47	38.42	48.44	10.02
	28	43.98 ± 7.62	43.82	37.71	49.78	12.07
	56	45.30 ± 7.82	45.23	38.80	50.61	11.81
	63	47.03 ± 8.11	47.72	41.13	53.07	11.94
	70	46.46 ± 7.88	46.55	40.52	52.43	11.91

Regression models were fitted for each individual HRQL domain, as well as for the PWB and EWB summary scores. For the E/E, A/C, C/R domains and summary scores, the best model was selected to be model 4 such that all components were included, with model 3 chosen for H/C. Table 4 shows the statistically significant results for the selected models, with most *p*-values being less than 0.001. The intercept terms represent the change in HRQL score over the first 14 days of the trial, with E/E and H/C showing greater changes than A/C and C/R. The slope terms for time show the changes over the remaining 56 days (days 14–70) of the trial with A/C now showing a slightly increased rate of change compared to E/E and C/R. For the PWB and EWB summary scores there were statistically significant results for both the intercept and time terms, showing an initial change and then a continued improvement during the remaining duration of the trial. The change in score from baseline (day 0) to the final time point at day 70 showed that the overall improvement in E/E and A/C was greater than the MID of 7 which indicates a clinically significant improvement in these domains over the duration of the trial. The 95% confidence intervals for the overall improvement in H/C and C/R included 7.

**Table 4:** Regression model results for 75 dogs with OA, treated with bedinvetmab.

HRQL factor	Intercept	Intercept *p*-value	Time component	Time *p*-value	8-week change	Overall change^a^
EE	6.69 (4.388, 8.995)	<0.001	0.079 (0.0421, 0.1159)	<0.001	4.42 (2.358, 6.490)	11.11 (6.746, 15.485)
HC	6.58 (4.676, 8.482)	<0.001				6.58 (4.676, 8.482)
AC	4.10 (2.693, 5.512)	<0.001	0.090 (0.0566, 0.1230)	<0.001	5.04 (3.170, 6.888)	9.14 (5.863, 12.400)
CR	3.02 (0.939, 5.092)	0.005	0.059 (0.0301, 0.0874)	<0.001	3.30 (1.686, 4.894)	6.32 (2.625, 9.986)
PWB	5.40 (3.749, 7.045)	<0.001	0.084 (0.0522, 0.1167)	<0.001	4.70 (2.923, 6.535)	10.10 (6.672, 13.580)
EWB	4.19 (2.475, 5.898)	<0.001	0.056 (0.0304, 0.0821)	<0.001	3.14 (1.702, 4.598)	7.33 (4.177, 10.496)

a Minimum important difference for domains E/E, H/C, A/C, C/R is 7.

#### 3.1.2 Cats

**Figure 5:**
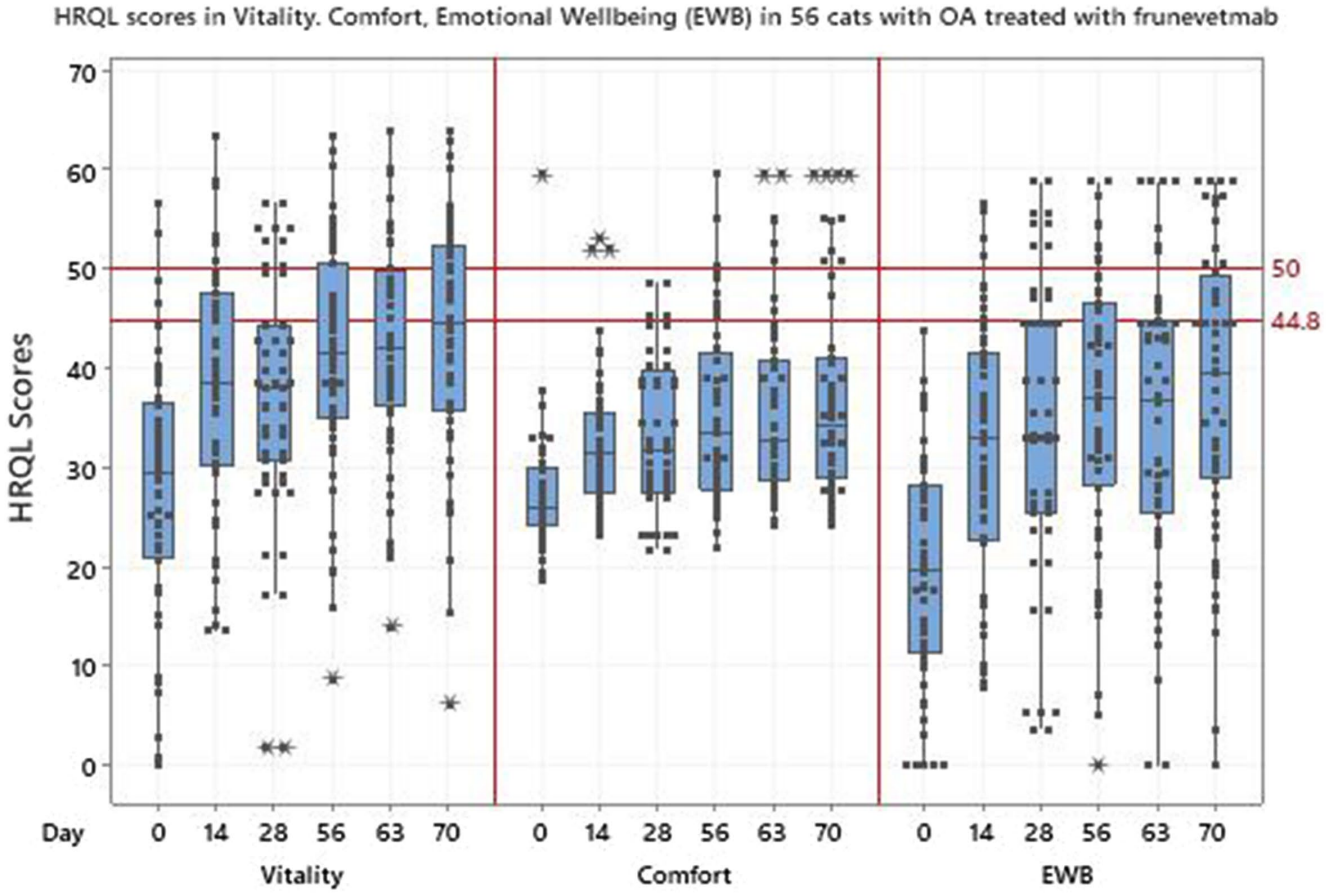
Scores in 3 domains of QOL-vitality, comfort, emotional wellbeing (EWB)-in 56 cats with osteoarthritis treated with frunevetmab.

Table 5 shows the HRQL descriptive statistics for the 56 cats treated with frunevetmab and Figure 5 shows the HRQL scores over time in all three domains. There was marked variability in the scores in all domains although this was less evident in the comfort domain. The most variability was seen in the EWB domain and the median score at day 0 was lowest in this domain, followed by comfort and then vitality. In no domain did the median scores reach the 44.8 threshold above which 70% of healthy cats will score.

**Table 5:** Descriptive statistics for scores in each of 3 domains of QOL (vitality, comfort and emotional wellbeing) at baseline (day 0, within 48 h of initial treatment with frunevetmab) and 5 subsequent time points to day 70 in 56 cats with osteoarthritis.

Domain	Day	Mean ± SD	Median	Q1	Q3	IQR
Vitality	0	28.21 ± 12.41	29.56	20.81	36.41	15.60
	14	38.16 ± 12.01	38.42	30.16	47.65	17.50
	28	37.26 ± 12.09	38.08	30.69	44.30	13.61
	56	41.18 ± 11.62	41.48	34.88	50.53	15.65
	63	42.41 ± 10.78	42.02	36.25	49.80	13.56
	70	43.38 ± 12.09	44.57	35.70	52.42	16.72
Comfort	0	27.18 ± 5.93	25.89	24.26	29.96	5.71
	14	32.33 ± 6.80	31.46	27.38	35.59	8.21
	28	33.29 ± 7.50	31.75	27.36	39.84	12.48
	56	35.23 ± 8.71	33.47	27.75	41.56	13.81
	63	35.72 ± 9.07	32.66	28.74	40.68	11.95
	70	37.17 ± 10.30	34.18	29.05	40.95	11.90
Emotional wellbeing (EWB)	0	19.58 ± 11.30	19.65	11.32	28.21	16.90
	14	31.51 ± 13.34	32.89	22.56	41.50	18.95
	28	32.87 ± 15.51	32.98	25.36	44.58	19.22
	56	36.14 ± 13.67	37.04	28.27	46.56	18.29
	63	34.76 ± 14.62	36.78	25.40	44.70	19.30
	70	37.80 ± 14.55	39.62	29.00	49.22	20.22
Physical well being	0	27.69 ± 8.33	28.79	22.14	32.10	9.96
	14	35.25 ± 8.91	34.75	29.49	41.41	11.92
	28	36.84 ± 10.0	36.23	30.25	45.59	15.34
	56	38.21 ± 9.49	37.73	32.01	45.91	13.90
	63	39.06 ± 9.29	38.65	33.08	44.38	11.30
	70	40.27 ± 10.53	39.19	34.29	46.98	12.69

**Figure 6:**
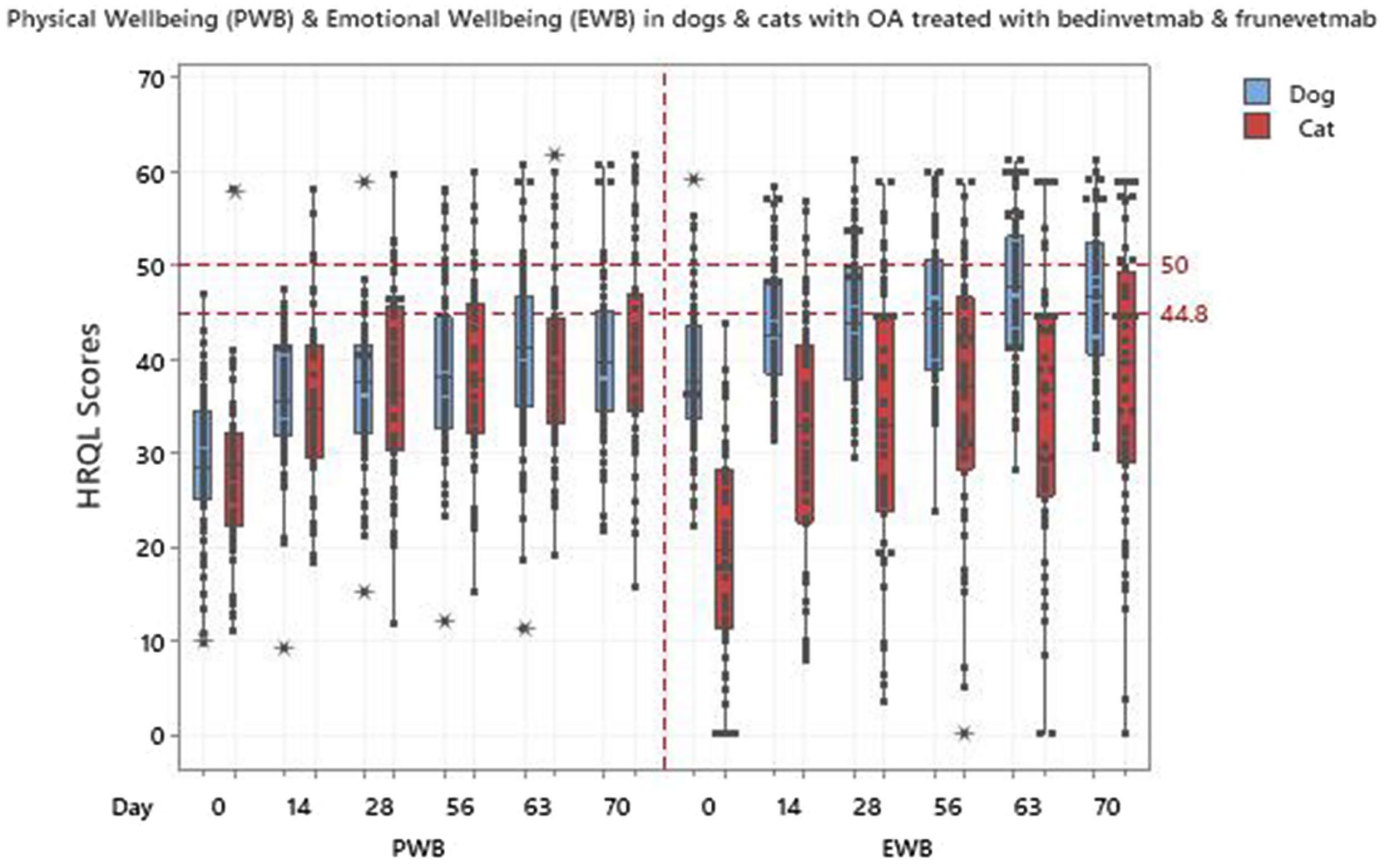
Physical wellbeing (PWB) and emotional wellbeing (EWB) in 75 dogs and 56 cats treated with bedinvetmab and frunevetmab respectively.

Regression models were fitted for each individual HRQL domain, as well as the PWB summary score. For the vitality and comfort domains and PWB the best model was selected to be model 4 such that all components were included, with model 3 chosen for EWB. Table 6 shows the statistically significant results for the selected model with all *p*-values being less than 0.001. The change for the first 14 days of the trial was greatest in the EWB domain and changes over the remaining 56 days of the trial were significant for vitality and comfort. The results for PWB were significant (*p*-values of <0.001) for both the intercept and time terms, showing an initial change and then a continued improvement during the remaining duration of the trial. The change in score from baseline (day 0) to the final time point at day 70 (Table 6) showed that the overall improvement in domain scores was greater than the MID of 5, 7.5 and 5 for vitality, comfort and EWB, respectively, which indicates a clinically significant improvement in all domains over the duration of the trial. Figure 6 shows how the cat PWB and EWB scores compare with those of the dog.

**Table 6:** Regression model results for 56 cats with OA, treated with frunevetmab.

HRQL factor	Intercept	Intercept *p*-value	Time component	Time *p*-value	8-week change	Overall change^a^
Vitality	8.63 (5.814, 11.435)	<0.001	0.088 (0.0428, 0.1331)	<0.001	4.93 (2.397, 7.454)	13.56 (8.21, 18.889)
Comfort	4.50 (2.545, 6.458)	<0.001	0.071 (0.0338, 0.1081)	<0.001	3.98 (1.893, 6.054)	8.48 (4.438, 12.512)
EWB	13.90 (10.696, 17.101)	<0.001				13.90 (10.696, 17.101)
PWB	6.56 (4.445, 8.680)	<0.001	0.079 (0.0419, 0.1170)	<0.001	4.42 (2.346, 6.552)	10.98 (6.791, 15.232)

a Minimum important difference for vitality, comfort, EWB is 5, 7.5, 5, respectively.

## 4 Discussion

### 4.1 Objective

Monoclonal antibodies represent a new class of OA pain medication for dogs and cats and as such published studies regarding their effectiveness are limited to their ability to decrease OA pain (22, 23). However, veterinary clinicians (10, 12), pet owners (42) and regulatory bodies are showing increasing interest in also demonstrating that therapeutics improve the QOL of treated animals. Accordingly, this study was undertaken to determine whether bedinvetmab and frunevetmab improved the QOL of dogs and cats with OA pain.

### 4.2 Population validity

Sampling in this study could best be described as convenience since veterinarians recruited any animals presenting at their clinic with OA for which their prescribed treatment was bedinvetmab (dogs) or frunevetmab (cats). Furthermore, this was an observational study, a form of exploratory clinical research commonly used in the medical and social science fields. However, to be considered scientifically robust, an observational study must have population validity which ensures that the results are generalizable to populations that share similar characteristics with the sample, in this case dogs and cats with OA. In that regard, dogs with OA tend to have age, breed, being overweight/obese and sex in common (4), whereas in cats there is less emphasis on breed and obesity has not been confirmed as a risk factor in cats (43–46).

Corral et al. (22) reported in a study of 287 dogs, 54% were female and 46% were male. This is in line with the finding in our study that there were more females than males. Conversely, Anderson et al. (4) reported that of 4,196 dogs with OA, there were more males than females, however this may not be generalizable to the general population of dogs with OA since this was a retrospective database review. These authors also reported that neutered individuals of either sex had almost twice the chance of being diagnosed with OA as those that were entire (4) and this did agree with our study in which 70% of dogs were neutered. Although in a study of 126 cats with OA, 74 cats were female and 52 were male (23) there are no large population-based studies to provide information on sex predilection in cats with OA and some evidence from small studies is conflicting (45, 46). Furthermore, Lascelles et al. (5) concluded that in a study of 100 cats where bodyweight, body condition score, sex and age were recorded, only age was significantly associated with the presence of DJD.

The age distribution of dogs and cats in this study supported the premise that OA can occur at any age but is more commonly identified in older animals. Although the prevalence of OA in the dog has been reported to be between 20 and 37% in dogs over 1 year (1–3), there is less detail reported for the cat. This is because feline studies have tended to investigate different age groups in hospital populations which are not generalizable to the OA population as a whole and report the incidence of degenerative joint disease (DJD) rather than OA *per se* (5, 6, 43, 45). Degenerative joint disease is an all-inclusive term that includes all degenerative pathology of a joint (47). Nevertheless, there is a consensus that OA is very common (48), with increasing identification as cats age (47). It is interesting to note that while the age distributions of both dogs and cats in this study support ageing as a major risk factor for the identification and treatment of OA, cats in this study tended to be older than dogs (mean 13.6 vs. 10.9; median 14 vs. 12). However, there appears to be a species difference regarding the age at which an animal becomes classed as senior or geriatric. According to the American Association of Feline Practitioners (AAFP) cats become senior from 11 years and geriatric from 15 years (49), whereas those values in dogs are 7 years and 12 years (50). On that basis our cat and dog samples were very similar, 88% senior dogs of which 59% were geriatric and 84% senior cats of which 53% were geriatric.

Although any breed of dog, including mixed breeds, can develop OA, large and giant-breed dogs are most often diagnosed. Within that category the Labrador Retriever, Golden Retriever, German Shepherd Dog, Rottweiler, Newfoundland, and Bernese Mountain Dogs have a hereditary predisposition because of their susceptibility to developmental orthopaedic disease (51). In this study it was impossible to determine the size of 16 dogs belonging to the crossbreed category and in 4 dogs the breed was unspecified. However, it is interesting that of the remaining 55 dogs 22 (40%) were classed as large and of these 20 (91%) were likely to have a hereditary disposition to OA-Labrador Retriever (16), Golden Retriever (3), Rottweiler (1). Furthermore, 56, 27 and 17% of our sample population were classified by weight as large (21–50 kg), medium (11–20 kg) and small (≤10 kg) dogs, respectively, which is in accordance with the findings of a large retrospective database review involving >16,000 dogs drawn from UK primary care practice which showed that one of the factors increasing the odds of having arthritis diagnosed was related to higher bodyweight (4).

Although being overweight/obese is an important risk factor for the development of OA in the dog (4, 52), in our study only body weight was documented which was insufficient to classify a dog as overweight (15% above optimal body weight) or as obese (30% above optimal body weight) (53) because the optimum weight range of individual dog breeds is generally expressed as a range. A definitive diagnosis would have required dimensional measurements and body condition scores (BCS) (54), neither of which are standard in primary care practice. With hindsight clinicians could have been asked for a subjective judgement regarding obesity which would have been valuable. The authors accept that this omission was a limitation of their study, but consider that the evidence presented regarding age, breed, bodyweight and sex in dogs and age in cats is sufficient to demonstrate the population validity of the study sample and its generalizability to the general population of dogs and cats with OA.

### 4.3 Quality of life measurement

Quality of life was measured using VetMetrica^™^ which is a web-based multidimensional generic HRQL tool that produces a profile of scores in several domains of HRQL, 4 for the dog and 3 for the cat. Whereas a single item score only demonstrates whether a subject is better or worse in response to treatment, a multidimensional tool that creates a profile of scores allows the researcher to interrogate these data to a greater extent and quantify where and by how much HRQL domains are changing in respect of each other. In the case of VetMetrica^™^ the creation of additional summary scores in emotional as well as physical wellbeing provides a robust way of identifying broader changes in the animal’s QOL, adding to the scope of the measurement which can be helpful in circumstances where an overview is desirable. In veterinary medicine the measurement of the emotional impact of disease and its treatment is a recent addition to the field of veterinary research, however it is one that has been recognised for decades in the human research arena. For example, the Medical Outcomes Study SF-36, published in 1992, is a well-established multidimensional generic HRQL tool that contains 8 domains which can be summarised into a mental component score (SF-MCS) and a physical component score (SF-PCS) (55, 56), the results of which have been used in many clinical trials including the investigation of monoclonal antibodies in human patients with arthritis (57, 58).

#### 4.3.1 Interpreting scores

The interpretation of the scores profile created by VetMetrica^™^ is original compared with other veterinary HRQL scales. Owner responses, scored as 0–6, are normalised to a mean score of 50 on a 1–100 scale, which represents that of the average healthy animal. This norm-based scoring and the establishment of a threshold at 44.8 in both species, (70% of healthy animals will lie above the threshold), provides the clinician with a simple method of ascertaining the health status of an individual dog or cat (39, 40) and makes the direct comparison between studies possible because they are all on the same numerical scale. Additionally, the results of investigations between species can be compared. This made it possible to evaluate the response to treatment with bedinvetmab in dogs compared to frunevetmab in cats.

#### 4.3.2 Outcome

Overall, there was marked variability in the HRQL scores for both dogs and cats which was not surprising given the heterogeneity of both populations used to collect owner responses to the assessments in a real - world situation. However, domains reflecting activity (A/C in dogs and Comfort in cats) exhibited less variability at day 0 and this could be indicative of the degree of pain associated with the OA before the onset of analgesia resulting from drug treatment. Corral et al. (22) and Gruen et al. (23) showed that at 7 days after treatment there was a detectable improvement in analgesia in dogs and cats, respectively, but although it is possible that this may have occurred earlier there is no supporting evidence available. Lacking this data, in this study the research team considered the change from baseline due to analgesic effect from drug administration before 48 h post treatment to be minimal and accordingly owners were given a deadline of 48 h after treatment by which time they were required to complete their first HRQL assessment.

It is interesting that at day 0, the physical well-being score was lower than that of emotional well-being in the dog, but the reverse was true of the cat. This may indicate a true species difference with respect to the effect of OA on QOL, or it may be a reflection of the fact that the cat population, being older than that of the dog, was suffering from more severe OA. However, perhaps a more likely explanation is that it was a consequence of other factors such as the influence of co-morbidities which are likely to be present in older dogs and cats. Compared with disease-specific tools, generic HRQL instruments are unique in that they measure the impact of all conditions affecting the animal and indeed are the only option when co-morbidities are present (33). It is noteworthy that the incidence of certain co-morbidities seems to be greater in cats with a primary diagnosis of OA than dogs. In a recent report produced by Banfield Pet Hospital (59) the results of a study population of 113,211 canine and 3,885 feline patients 12 months post initial diagnosis of OA showed the following, 1.1% of the dog population had a diagnosis of diabetes mellitus compared with 3.7% of cats; 3.1% of dogs had thyroid disease compared with 9.8% of cats and 1.4% of dogs had chronic kidney disease compared with 19.4% of cats. These are medical conditions that may have a significant impact on EWB in animals as they do in people (60–62), and accordingly, if we assume a similar incidence of these in the real-world populations used in this study, this could account for the comparatively low EWB scores in the cat as well as the greater variability seen in the cat EWB scores compared with the dog.

In the dog, from day 56 onwards, median scores in EWB reached the threshold of 44.8 (70% of healthy dogs will lie above the threshold), indicating the noticeable ability of the treatment to make the dog “feel better.” By comparison, the cat median scores improved although they remained below this threshold. This may reflect the low starting point for EWB scores, but nevertheless the pattern of improvement in the cat mirrored that of the dog and was no less remarkable. In contrast, the improvement in PWB was such that no median scores in either species reached the 44.8 threshold. This is not surprising given the fact that treatment alleviates the pain associated with OA and therefore improves physical functioning but does not change the underlying joint pathology and is not sufficient to restore the animal back to a normal physical well-being.

For formal analysis HRQL data were analysed using a mixed-effect model with intercept and time as fixed effects and the dog or cat ID as a random, with model selection via BIC then used to determine if all terms should be included. The full model was used for all domains and summary scores with the exceptions of H/C for the dog and EWB for the cat, where no time component was selected using BIC and therefore this was not included in the models. A possible explanation for this might be that there is a temporal difference between the improvement in emotional and physical wellbeing. We have reported this previously in a dog with OA which showed a rapid improvement in EWB after acupuncture and life-style changes, peaking at approximately 1 month whereas in PWB that point was not reached until month 11 (39). Similarly, this may be the case in this study with most of the emotional improvement, represented in large part by the domain H/C in the dog and EWB in the cat, occurring in the first 2 weeks with little increase after that, hence no time effect according to BIC. Conversely, in the physical domains only a modest physical improvement takes place in the first 2 weeks with most occurring over the remainder of the study, allowing for a model that includes time effect.

For the improvement in HRQL score from day 0 to day 14, all domains and summary scores were statistically significant at the 5% significance level. Although this initial improvement showed unconditionally that bedinvetmab and frunevetmab markedly improved the QOL of dogs and cats with OA, the fact that improvement was shown to continue for the duration of the trial provided additional evidence for its effectiveness.

#### 4.3.3 Responsiveness

In general, the responsiveness of a measurement instrument is defined as “the extent to which an instrument can measure change when change has occurred” (63) and refers to the validity of a score change (64). In healthcare, while the demonstration of a change that is statistically significant is important, identifying a change that is clinically significant is often regarded as more relevant. The minimum important difference (MID) is a means of quantifying responsiveness and has been determined for both dog and cat VetMetrica^™^ tools in populations suffering from unspecified chronic conditions (38, 39). In this study the change scores from day 0 to day 70 (overall change) in all cat domains exceeded the MID indicating clinically significant improvement. The same applied to E/E and A/C in the dog, but the domains H/C and C/R narrowly missed the threshold. However, the MID is not an intrinsic property of a scale but depends on the clinical context in which the scale is used. Accordingly, the MIDs reported here may not be accurate when the scale is used in specific disease populations like OA. For example, in people, the use of the MOS SF-36 has been reported in disease specific populations such as Crohn’s Disease (65), OA (35), cardiac disease (66) as well as asthma (67) and the MID varies considerably between these conditions. On that basis the overall scores for H/C and C/R may also be clinically significant, especially since the confidence intervals for the median included the MID value 7.

## 5 Value

The importance of this investigation to the veterinary community has been defined in the introduction, but the significance to the owner is also noteworthy. As a result of the strong human animal bond, pets are often regarded as part of the family (68, 69) and so not surprisingly their QOL is important to the owner. Indeed Hale et al. (42) concluded that in the UK “Owners want to talk about holistic dog care,” and of 410 dog owners surveyed, 95.8% were comfortable discussing their dog’s QOL with their vet. Furthermore, a high proportion of owners (70.8%) expressed interest in using assessment tools to measure QOL. In a market research study conducted in 2021, 92% of 266 dog owners in the US awarded the top ratings to “improved QOL” when asked to consider a list of attributes of a therapeutic designed to treat chronic kidney disease and this also applied to 86% of 144 owners in the UK.^6^

## 6 Conclusion

Using data collected in an observational study based in primary care practices, the results of which were shown to be generalisable to the general population of dogs and cats with OA, this study has produced RWE to demonstrate that bedinvetmab and frunevetmab improve the QOL in dogs and cats, respectively. The authors consider that this latest evidence for the use of these therapeutics could be useful in the clinical management of osteoarthritis pain and in post-marketing clinical trials. Equally valuable is the fact that the pet owner can be confident in the knowledge that not only is their pet’s mobility improved when treated for OA with these medications, but they feel much better too.

## Data availability statement

The original contributions presented in the study are included in the article/Supplementary material, further inquiries can be directed to the corresponding author.

## Ethics statement

The animal studies were approved by Zoetis Ethical Review Board. The studies were conducted in accordance with the local legislation and institutional requirements. Written informed consent was obtained from the owners for the participation of their animals in this study.

## Author contributions

JR: Methodology, Writing – original draft, Writing – review & editing, Project administration. EG: Writing – review & editing, Conceptualization, Data curation, Methodology, Project administration, Supervision. VD: Formal analysis, Writing – review & editing. JT: Project administration, Software, Supervision, Writing – review & editing. MS: Formal analysis, Validation, Writing – review & editing.

## Footnotes

^1^MARC Veterinarian Attitude and Usage Market Research Study 2021.

^2^Committee for Medicinal Products for Veterinary Use. (2020). EMA Librela—Summary of Opinion. EMA/CVMP/438128/2020. Available online at: https://www.ema.europa.eu/en/medicines/veterinary/EPAR/librela.

^3^Committee for Medicinal Products for Veterinary Use. (2020). EMA Solensia— Summary of Opinion. EMA/CVMP/631469/2020. Available online at: http://www.ema.europa.eu/en/medicines/veterinary/summaries-opinion/solensia.

^4^Penn Vet|CBPI Tool (upenn.edu).

^5^ www.minitab.com

^6^Zoetis Pet Owner CKD A&U (attitudes and use) market research studies 2021.
