# Supplementary material for: Measuring the effect of the anti-nerve growth factor antibodies bedinvetmab and frunevetmab on quality of life in dogs and cats with osteoarthritis using a validated health-related quality of life outcome measure: an observational real-world study
Source: Front Vet Sci. 2024 Aug 14;11:1395360. doi: 10.3389/fvets.2024.1395360 (PMC11349630; doi:10.3389/fvets.2024.1395360)
Supplement: Supplementary file 1 [file Data_Sheet_1.pdf]

VetMetrica Feline HRQL

IMPORTANT INFORMATION

VetMetrica™ for cats is an online tool which provides a profile of scores in 3 domains of QOL – Vitality, Comfort and Emotional Wellbeing and these scores are generated automatically and instantaneously by a coded algorithm embedded in the Zoetis server. **Simply adding up the owner responses to each item will not generate a valid HRQL score.**

If you would like access to VetMetrica™ please use the contact us tab at www.vetmetrica.com

Cat identification number

Copyright

Instructions

Welcome to the VETMETRICA Assessment for your cat.

Please take a few minutes to tell us how your cat acts and feels.

- Please do not think too long about your answer.
- You may find it helpful to read aloud the ends of the scale.
- Using a black pen, please clearly fill in the circle that best describes how your cat acts and feels.

Please tell us how well this word describes your cat as he/she is today - Active

	0	1	2	3	4	5	6	
Not at all active	◯	◯	◯	◯	◯	◯	◯	Couldn't be more active

Please tell us how well this word describes your cat as he/she is today - Unsteady

	0	1	2	3	4	5	6	
Not at all unsteady	◯	◯	◯	◯	◯	◯	◯	Couldn't be more unsteady

Please tell us how well this word describes your cat as he/she is today - Energetic

	0	1	2	3	4	5	6	
Not at all energetic	◯	◯	◯	◯	◯	◯	◯	Couldn't be more energetic

Please tell us how well this word describes your cat as he/she is today - Comfortable

	0	1	2	3	4	5	6	
Not at all comfortable	◯	◯	◯	◯	◯	◯	◯	Couldn't be more comfortable

Please tell us how well this word describes your cat as he/she is today - Lethargic

	0	1	2	3	4	5	6	
Not at all lethargic	◯	◯	◯	◯	◯	◯	◯	Couldn't be more lethargic

Please tell us how well this word describes your cat as he/she is today – Showing hunting behaviour

	0	1	2	3	4	5	6	
Not showing hunting behaviour at all	◯	◯	◯	◯	◯	◯	◯	Couldn't be showing hunting behaviour more

Please tell us how well this word describes your cat as he/she is today - Lively

	0	1	2	3	4	5	6	
Not at all lively	◯	◯	◯	◯	◯	◯	◯	Couldn't be more lively

Please tell us how well this word describes your cat as he/she is today - Alert

	0	1	2	3	4	5	6	
Not at all alert	◯	◯	◯	◯	◯	◯	◯	Couldn't be more alert

Please tell us how well this word describes your cat as he/she is today - Sore

	0	1	2	3	4	5	6	
Not at all sore	◯	◯	◯	◯	◯	◯	◯	Couldn't be more sore

Please tell us how well this word describes your cat as he/she is today - Content

	0	1	2	3	4	5	6	
Not at all content	◯	◯	◯	◯	◯	◯	◯	Couldn't be more content

Copyright

Please tell us how well this word describes your cat as he/she is today - Playful

Not at all playful 0 ○ 1 ○ 2 ○ 3 ○ 4 ○ 5 ○ 6 ○ Couldn't be more playful

Please tell us how well this word describes your cat as he/she is today – Uncomfortable

Not at all uncomfortable 0 ○ 1 ○ 2 ○ 3 ○ 4 ○ 5 ○ 6 ○ Couldn't be more uncomfortable

Please tell us how well this word describes your cat as he/she is today – Enjoying the thing he/she usually does

Not enjoying the things he/she usually does at all 0 ○ 1 ○ 2 ○ 3 ○ 4 ○ 5 ○ 6 ○ Couldn't be enjoying the things he/she usually does more

Please tell us how well this word describes your cat as he/she is today - Jumping or climbing up/down as usual

Not jumping or climbing up/down as usual at all 0 ○ 1 ○ 2 ○ 3 ○ 4 ○ 5 ○ 6 ○ Jumping or climbing up/down as usual

Please tell us how well this word describes your cat as he/she is today - Exploring

Not exploring at all 0 ○ 1 ○ 2 ○ 3 ○ 4 ○ 5 ○ 6 ○ Couldn't be exploring more

Please tell us how well this word describes your cat as he/she is today - Feeling himself/herself

Copyright

	0	1	2	3	4	5	6	
Not feeling himself/herself at all	◯	◯	◯	◯	◯	◯	◯	Couldn't be feeling more himself/herself

Please tell us how well this word describes your cat as he/she is today - Stiff

	0	1	2	3	4	5	6	
Not at all stiff	◯	◯	◯	◯	◯	◯	◯	Couldn't be more stiff

Please tell us how well this word describes your cat as he/she is today - Happy

	0	1	2	3	4	5	6	
Not at all happy	◯	◯	◯	◯	◯	◯	◯	Couldn't be more happy

Please tell us how well this word describes your cat as he/she is today - Inquisitive

	0	1	2	3	4	5	6	
Not at all inquisitive	◯	◯	◯	◯	◯	◯	◯	Couldn't be more inquisitive

Please tell us how well this word describes your cat as he/she is today - Slow

	0	1	2	3	4	5	6	
Not at all slow	◯	◯	◯	◯	◯	◯	◯	Couldn't be more slow

Copyright
